# Surface Area Loss and Increased Sphericity Account for the Splenic Entrapment of Subpopulations of *Plasmodium falciparum* Ring-Infected Erythrocytes

**DOI:** 10.1371/journal.pone.0060150

**Published:** 2013-03-28

**Authors:** Innocent Safeukui, Pierre A. Buffet, Sylvie Perrot, Alain Sauvanet, Beatrice Aussilhou, Safi Dokmak, Anne Couvelard, Dominique Cazals Hatem, Narla Mohandas, Peter H. David, Odile Mercereau-Puijalon, Geneviève Milon

**Affiliations:** 1 Institut Pasteur, Immunologie Moléculaire des Parasites, Département de Parasitologie Mycologie, Paris, France; 2 CNRS, URA2581, Paris, France; 3 Center for Rare and Neglected Diseases, University of Notre Dame, Notre Dame, Indiana, United States of America; 4 INSERM - UPMC (Paris 6 University) UMRs945, Paris, France; 5 AP-HP, Department of Parasitology, Pitié Salpétrière Hospital, Paris, France; 6 Department of Surgery, Beaujon Hospital, AP-HP, Clichy, France; 7 Department of Pathology, Beaujon Hospital, AP-HP, Clichy, France; 8 New York Blood Centre, New York, New York, United States of America; 9 Institut Pasteur, Immunophysiologie et Parasitisme, Département de Parasitologie Mycologie, Paris, France; Weill Cornell Medical College, United States of America

## Abstract

*Ex vivo* perfusion of human spleens revealed innate retention of numerous cultured *Plasmodium falciparum* ring-infected red blood cells (ring-iRBCs). Ring-iRBC retention was confirmed by a microsphiltration device, a microbead-based technology that mimics the mechanical filtering function of the human spleen. However, the cellular alterations underpinning this retention remain unclear. Here, we use ImageStream technology to analyze infected RBCs’ morphology and cell dimensions before and after fractionation with microsphiltration. Compared to fresh normal RBCs, the mean cell membrane surface area loss of trophozoite-iRBCs, ring-iRBCs and uninfected co-cultured RBCs (uRBCs) was 14.2% (range: 8.3–21.9%), 9.6% (7.3–12.2%) and 3.7% (0–8.4), respectively. Microsphilters retained 100%, ∼50% and 4% of trophozoite-iRBCs, ring-iRBCs and uRBCs, respectively. Retained ring-iRBCs display reduced surface area values (estimated mean, range: 17%, 15–18%), similar to the previously shown threshold of surface-deficient RBCs retention in the human spleen (surface area loss: >18%). By contrast, ring-iRBCs that successfully traversed microsphilters had minimal surface area loss and normal sphericity, suggesting that these parameters are determinants of their retention. To confirm this hypothesis, fresh normal RBCs were exposed to lysophosphatidylcholine to induce a controlled loss of surface area. This resulted in a dose-dependent retention in microsphilters, with complete retention occurring for RBCs displaying >14% surface area loss. Taken together, these data demonstrate that surface area loss and resultant increased sphericity drive ring-iRBC retention in microsphilters, and contribute to splenic entrapment of a subpopulation of ring-iRBCs. These findings trigger more interest in malaria research fields, including modeling of infection kinetics, estimation of parasite load, and analysis of risk factors for severe clinical forms. The determination of the threshold of splenic retention of ring-iRBCs has significant implications for diagnosis (spleen functionality) and drug treatment (screening of adjuvant therapy targeting ring-iRBCs).

## Introduction

The dynamic changes in the mechanical properties of red blood cells (RBCs) following invasion by the malaria parasite, *Plasmodium falciparum*, is a major determinant of the passage of infected RBCs (*Pf-*iRBCs) through the microvascular bed and the inter-endothelial slits (IES) of the splenic red pulp venous sinus. RBCs harboring young ring forms (ring-iRBCs) are the only asexual intraerythrocytic stage seen in the peripheral circulation, implying that infected RBCs at this early stage of parasite development are the only ones that have the ability to traverse the microvascular bed and the splenic red pulp. However, e*x vivo* perfusion of human spleens with *in vitro* cultured parasite infected RBCs showed that even at early stages of development about 50% of ring-iRBCs were retained in the spleen [1]. Ring-iRBCs accumulated upstream from IES of the venous sinuses [1], a spleen-specific micro-anatomic structure known to retain RBCs with reduced deformability [2], [3], [4], [5]. Similar extents of ring-iRBC retention were observed in the microsphiltration device, a system previously shown to mimic the geometry of narrow and short IES of the splenic sinuses [5]. This suggests heterogeneity of ring-iRBCs with regard to their mechanical properties that may account for their differential splenic entrapment. However, the cellular modifications underpinning the deformability defect and the splenic retention of ring-iRBCs remain to be defined.

RBC deformability is influenced by membrane viscoelasticity, intracellular viscosity and cell geometry, particularly the surface area – to – volume (S/V) ratio [6], [7], [8]. These parameters are altered in *Pf-*iRBCs [9], [10], [11]. Based on micropipette aspiration studies, decreased S/V ratio was implied as a contributor to the decreased deformability of ring-iRBCs; however, the extent of decreased deformability was deemed unlikely to hinder circulation of ring-iRBC [9]. In contrast, the finding of increased volume and reduced surface area of ring-iRBCs using a microfluidic device was, however, interpreted as a possible contributor to their splenic retention [10]. On the other hand, quantitative imaging using fluorescence confocal microscopy led Esposito and colleagues to conclude that the *Pf-*iRBC volume remains essentially constant throughout the asexual parasite development [11]. These investigators also noted that while ring-iRBCs showed only minimal changes in membrane surface area compared to uninfected co-cultured RBCs (uRBCs), there was a significant membrane area loss with progressing intracellular maturation of the parasite to trophozoite and schizont stages [11].

In the present study, we explored changes in morphology and dimensions of *Pf-*iRBCs with ImageStream technology [8]. One of the principal advantages of this technology is that in contrast to micropipette techniques or microfluidic devices or quantitative imaging using fluorescence confocal microscopy where the number of cells analyzed is limited to less than a few hundred cells at best, a very large number of cells can be analyzed (thousands of cells). We compared the morphological characteristics of ring-iRBCs before and after microsphiltration, a recently developed technology, which challenges cell deformability in a way mimicking the mechanical sensing of the human spleen [5]. We show that the combination of a reduced surface area and resultant increased sphericity is the main contributor to the reduced deformability of ring-iRBCs that results in their retention in the microsphilters. These findings documenting splenic entrapment of subpopulations of ring-iRBCs have implications for the estimation of parasite burden in infected individuals based on the observed parasitemia in peripheral blood.

## Results

### *P. falciparum* Ring-infected Erythrocytes Display Surface Area Loss and Increased Sphericity Leading to their Reduced Deformability

**Figure 1:**
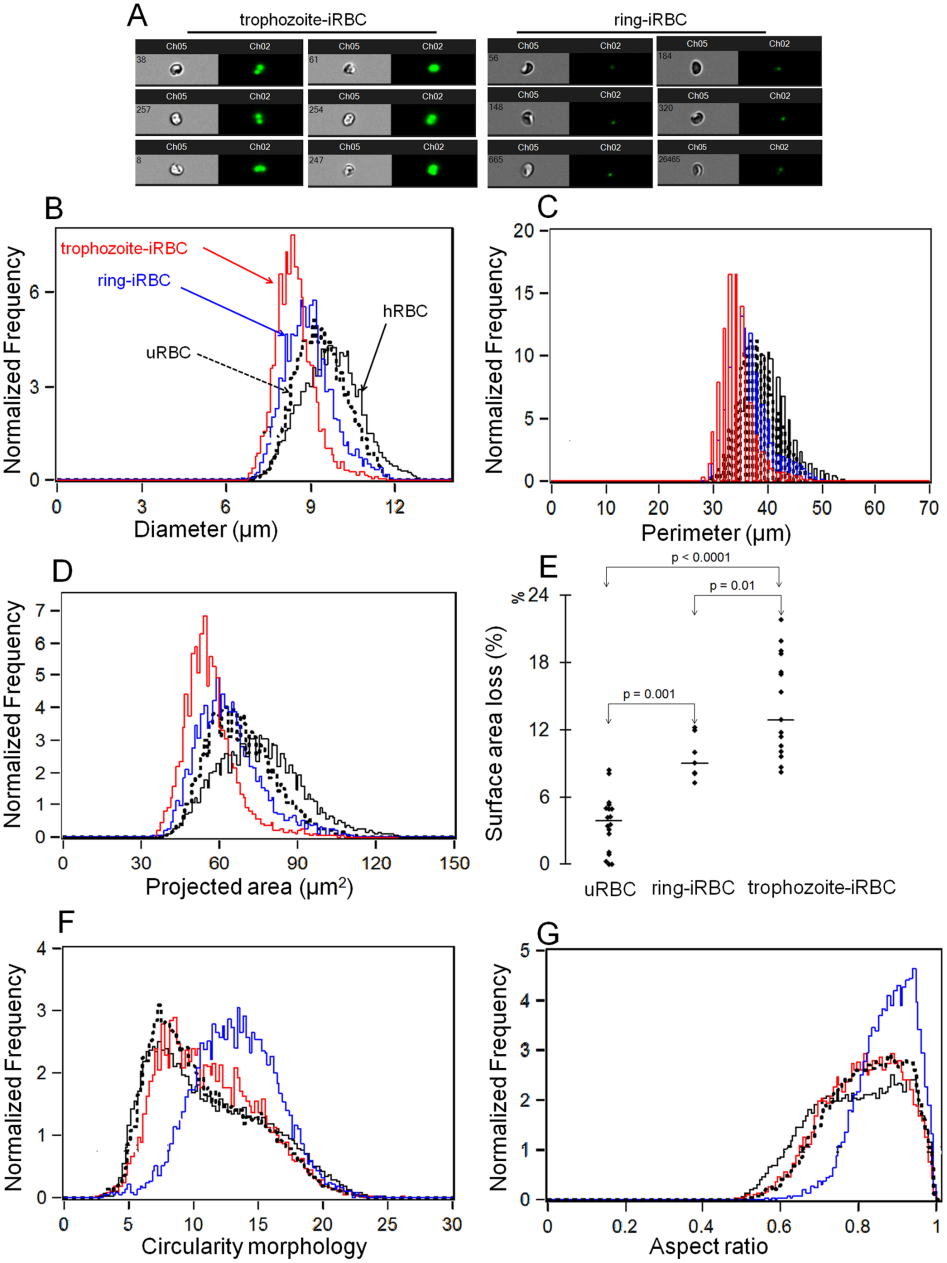
Geometry and functional alterations of uninfected or *P. falciparum* infected red blood cells. Analysis of uninfected (uRBCs or hRBCs) or infected (ring-iRBCs, age <9 hours; or trophozoite-iRBCs, age: 30 to 35 hours) cell dimension and morphology using ImageStream technology (**A–G**). Before acquisition, parasite nucleus was labeled with Hoechst 33342 (diluted 1∶1000; Invitrogen, Carlsbad, CA; LSR; BD Biosciences, San Jose, CA), allowing differentiation of infected from uninfected co-culture RBCs. For each population of *Pf*-iRBCs, more than 10000 images were collected and analyzed with ImageStream technology. Captured images of infected RBCs are displayed in (**A**). There is a shift toward lower values of the distribution of iRBC diameter, perimeter and projected surface area, which increases with parasite maturation (**B–D**). Ring-iRBCs display a more pronounced surface area loss than uninfected co-culture RBCs (uRBC), but smaller than trophozoite-iRBCs (**E**). Circularity morphology and/or aspect ratio of iRBCs were increased compared to healthy RBCs (hRBCs) (**F–G**). The surface area loss of *Pf*-iRBCs or uRBCs = [1– (mean values of projected surface area of iRBCs or uRBCs/mean values of projected surface area of hRBC)]*100.

**Figure 2:**
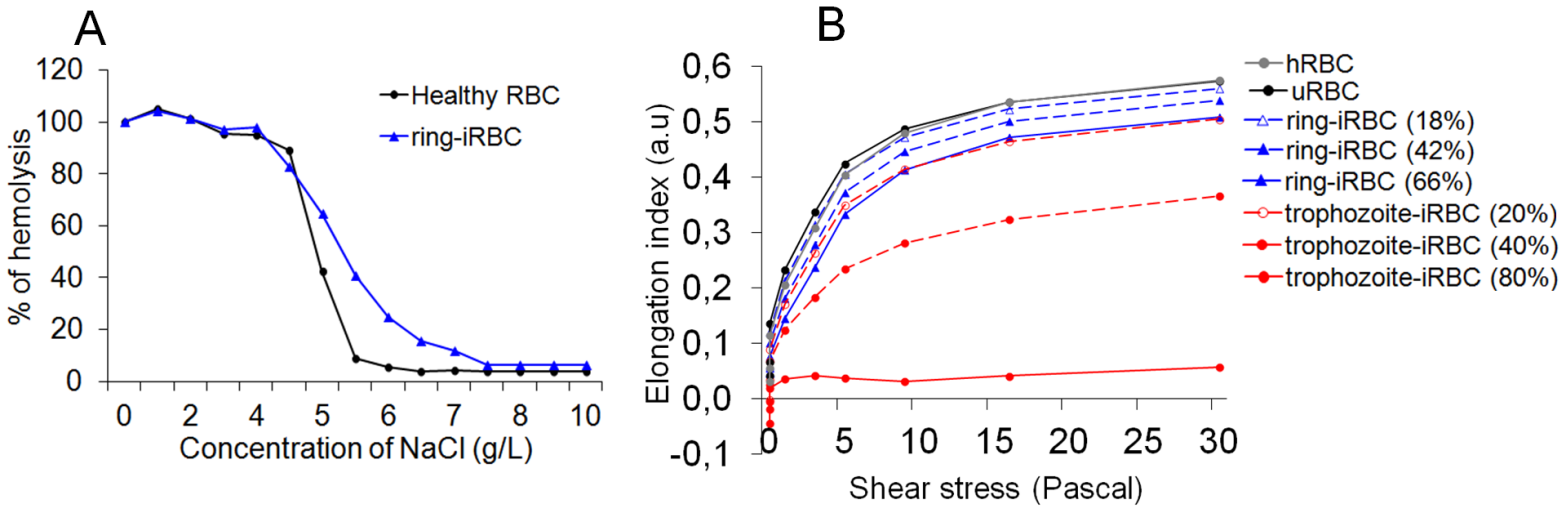
Osmotic fragility and deformability of uninfected or *P. falciparum* infected red blood cells. Osmotic fragility of a population of ring-iRBCs with 49% parasitemia was slightly decreased (**A**). Ektacytometer analysis showed that trophozoite-iRBCs were totally rigid, while ring-iRBCs were moderately, but significantly less deformable than control RBCs (uRBCs or hRBCs) (**B**).

The dimensions (diameter, perimeter and projected surface area) and morphological parameters (circularity and aspect ratio) of ring-iRBCs (<9 hours of age) were analyzed with ImageStream technology and compared to control RBCs (uRBCs – uninfected RBCs from the same culture or hRBCs – uncultured RBCs from the same donor). We also analyzed for the purpose of comparison trophozoite-iRBCs (30 to 35 hours of age) from the same culture (Figure 1A–G). Compared to hRBCs, there was a shift toward lower diameter, perimeter and projected surface area values of uRBCs and of *Pf*-iRBCs, which became more marked as the parasite matured (Figures 1B–D). The mean and range of surface area loss of ring-iRBCs (9.6%, 7.3–12.2%) were higher than uRBCs (3.7%, 0–8.4%, p = 0.001), but lower than trophozoite-iRBCs (14.2%, 8.3–21.9%, p = 0.01) (Figure 1E). There was a marginal shift toward higher values of circularity and aspect ratio (sphericity) for uRBCs compared to hRBCs, and further increases in their values for ring-iRBCs (Figure 1F–G). Circularity and aspect ratio values were similar between uRBCs and trophozoite-iRBCs (Figure 1F–G). RBCs with decreased S/V ratio and increased sphericity [7], [8] are osmotically fragile. An increase in osmotic fragility of a population of ring-iRBCs (tested at 49% parasitemia) could be documented (Figure 2A). Together, these results support the conclusion that the ring-iRBCs have a reduced surface area with a slightly increased sphericity (i.e reduced S/V ratio) and as a result are osmotically fragile.

The deformability changes of hRBCs, uRBCs and *Pf*-iRBCs sampled at different parasitemia (16–66% for ring-iRBCs and 20–80% for trophozoite-iRBCs) were analyzed by ektacytometry, which quantifies the extent of cell deformation as a function of applied shear stress (Figure 2B). The elongation index (EI) was similar between uRBCs and hRBCs (Figure 2B). As expected, trophozoite-iRBCs had a very low EI at all levels of applied shear stresses (Figure 2B), probably reflecting increased membrane rigidity, altered intracellular viscosity and the presence of a large, rigid parasite within the host RBC cytoplasm [9]. Ring-iRBCs were moderately, but significantly less deformable than uRBCs or hRBCs, with a reduced maximum value of EI at 66% parasitemia and parallel plateaus at high applied shear stress values (Figure 2B). These deformability profiles are characteristic of RBCs with decreased membrane surface area and reduced S/V ratio [7], [8], strongly suggesting that loss of surface area combined with increased sphericity (i.e reduced S/V ratio) is a major contributor to the reduced deformability of ring-iRBCs.

### Retention of *P. falciparum* Ring-infected Erythrocytes in Microsphilters is Linked to their Reduced Surface Area and Increased Sphericity

**Figure 3:**
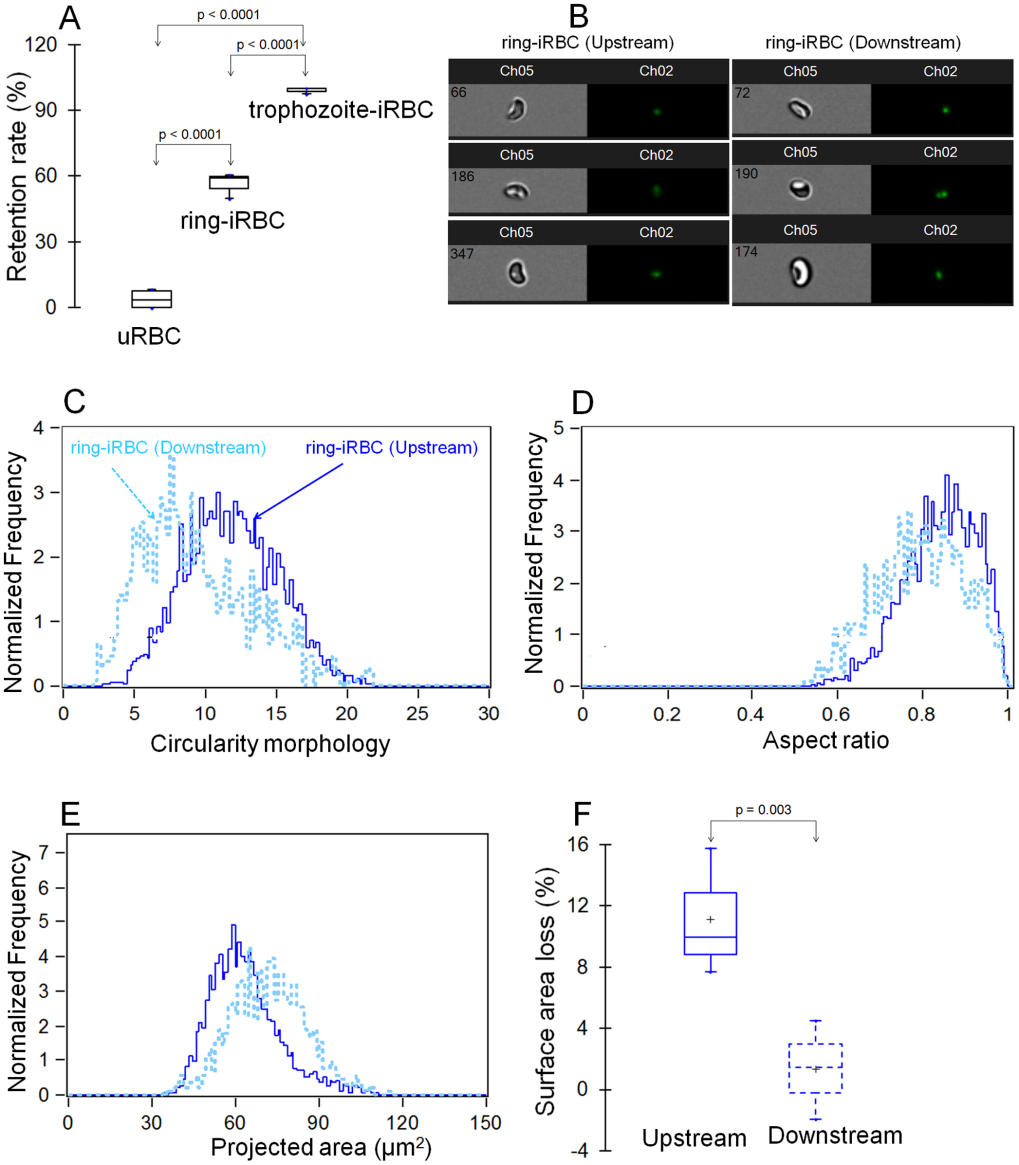
Retention of ring-iRBCs in microsphilters is linked to surface area loss associated with increased sphericity. Retention of uRBCs or RBCs hosting ring (age <9 hours) or trophozoite (age: 30 to 35 hours) forms of *P. falciparum* in the microspheres (**A**) (mean of 4 independent experiments). Analysis with ImageStream technology of the circularity, aspect ratio (sphericity) and projected surface area of unfractionated (Usptream) or flow through (Downstream) ring-iRBCs from microspheres (**B–F**) (shown is a representative experiment out of 3). For each population of ring-iRBCs, more than 1000 images were collected and analyzed with ImageStream technology. Captured images of upstream and downstream ring-RBCs are displayed in (**B**). There is a shift toward normal values of morphological and dimension parameters of flow-through ring-iRBCs (**C–F**). Surface area loss of unfractionated (upstream) or flow through (downstream) ring-iRBCs (**F**) (n = 3 independent experiments). The surface area loss of upstream or downstream ring-iRBCs = [1– (mean values of projected surface area of upstream or downstream ring-iRBCs/mean values of projected surface area of hRBC)]*100.

Microsphiltration showed that whereas uRBCs readily flowed through the column with only a very small fraction of cells being retained (mean ± standard deviation of retention rate: 3.8±3.6%), trophozoite-iRBCs were unable to transit through the column of microspheres with 98.8±1.2% cell retention (Figure 3A). The retention rate for ring-iRBCs (<9 hours of age) was intermediate (56.4±5.9%) between uRBCs and trophozoite-iRBCs (p<0.0001 for both trophozoite-iRBCs and ring-iRBCs compared to uRBCs, or for trophozoite-iRBCs compared to ring-iRBCs) (Figure 3A). These results are consistent with previous microsphiltration findings [5] and ex *vivo* perfusion studies using human spleens [1], [5], and confirm the heterogeneity of ring-iRBC populations with regard to their ability to traverse narrow pores.

The cell shape and size of uRBCs and ring-iRBCs before and following filtration though the microspheres in 3 independent experiments were analyzed with ImageStream technology (Figure 3B–F). Unfractionated (upstream from microsphilters) uRBCs, flow-though (downstream from microsphilters) uRBCs and flow-through ring-iRBCs display similar distributions of circularity, aspect ratio and projected surface area values (data not shown). However, the flow-through ring-iRBCs had lower values of circularity and aspect ratio compared to the unfractionated ring-iRBCs (mean ± standard deviation of sphericity increase: 0.6±3.4% *vs* 4.9±1.8%; p = 0.02) (Figure 3C–D). The flow-through ring-iRBCs had minimal surface area loss compared to the unfractionated ring-iRBCs (mean±sd: 1.3±2.0% *vs*. 11.1±1.2%; p = 0.003) (Figure 3E–F). The similar projected surface area and aspect ratio values of flow-through ring-iRBCs and uRBCs, both significantly different from the unfractionated ring-iRBCs (Figure 3E) indicate that ring-iRBCs with decreased projected surface area and increased sphericity could not traverse column of microspheres. Based on the findings that 56±6% of ring-iRBC were retained in the column of microspheres and a documented average surface area loss of 11.1%(±1.2%) in unfractionated ring-iRBCs and normal projected surface area of flow-through ring-iRBCs, we estimate that retained ring-iRBCs have lost on average 17% (range: 15–18%) of their surface area. Taken together, these results suggest that the retention of ring-iRBCs in microsphilters is primarily attributable to their reduced surface area and consequent increased sphericity.

**Figure 4:**
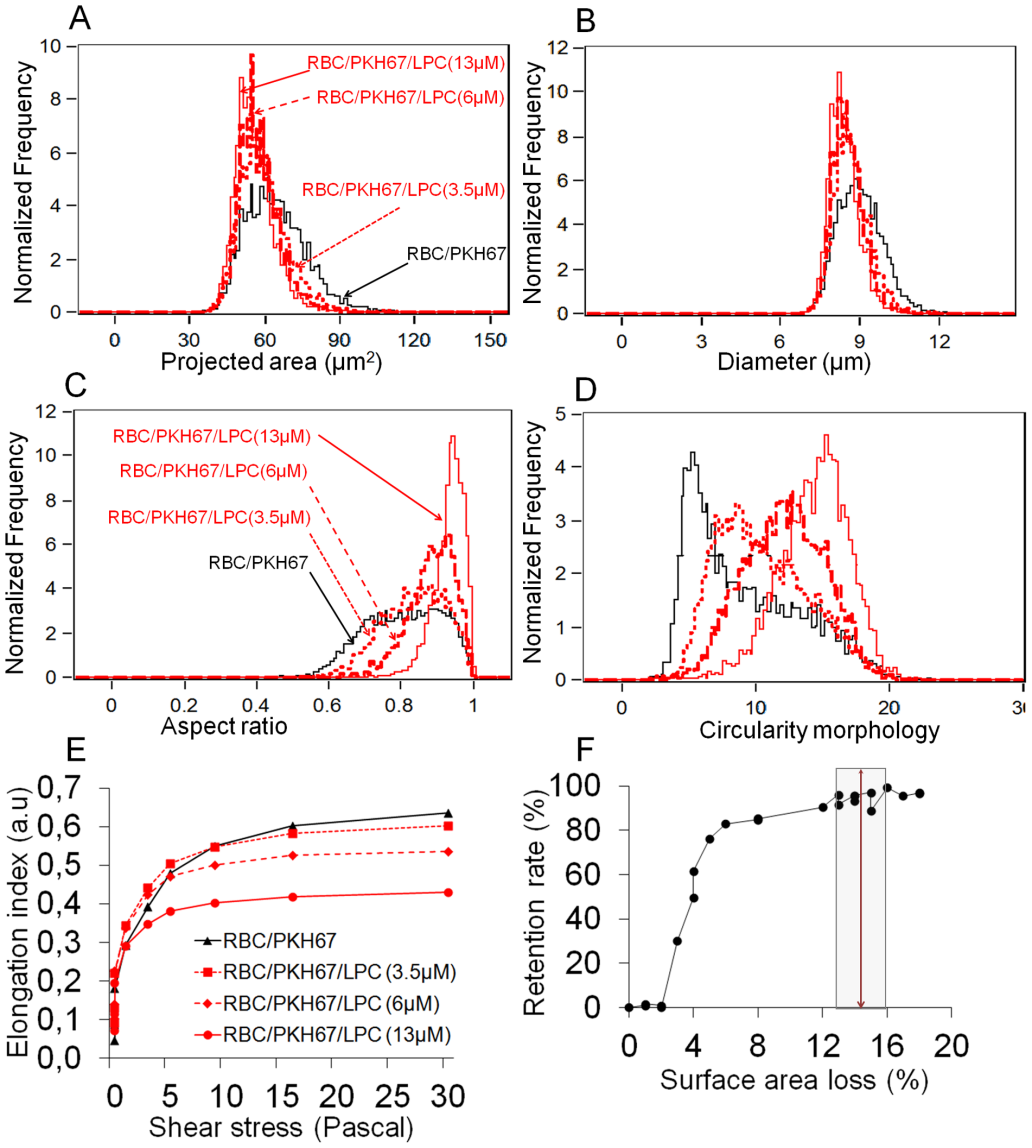
Retention of LPC-treated RBCs in microsphilters increases with the degree of surface area loss and sphericity. Analysis of LPC-treated RBC projected surface area, diameter, aspect ratio and circularity using ImageStream technology (**A–D**) (shown is one representative experiment out of 4). For each population of RBCs (LPC-treated RBCs or untreated control), at least 10000 cells were collected and analyzed with ImageStream technology. There is a concentration-dependent shift toward lower values of the distribution of projected surface area (**A**) and diameter (**B**). Aspect ratio (**C**) and circularity (**D**) of LPC-treated RBCs increase with the degree of surface area loss. LPC treatment results in dose-dependent reduction of RBC deformability measured by Ektacytometer analysis (**E**) (one representative experiment out of 4). Linear regression fit of the correlation between surface area loss and the level of LPC-treated RBCs retention in the microspheres (**F**) (n = 4 independent experiments). The surface area loss of LPC-treated RBCs = [1– (mean values of projected surface area of LPC-treated RBCs/mean values of projected surface area of hRBC)]*100.

Given that both membrane viscoelasticity and cytoplasmic viscosity affect the deformability of ring-iRBCs [9], their additional contribution to the mechanical entrapment of ring-iRBCs in the microsphilters could not be totally excluded. Therefore, a controlled loss of surface area associated with increased sphericity was induced in hRBCs by *in vitro* exposure to LPC, reported to influence RBC sphericity [8], S/V ratio [7], [8], [12], [13], [14] and rigidity [6], [7], [8], [15], but not membrane viscoelasticity [6], [7], [15] or cytoplasmic viscosity [7], [8]. Confirming previous findings [8], ImageStream analysis showed that exposure of hRBC to LPC resulted in a dose-dependent decrease in cell projected surface area and diameter (Figure 4A–B), increased aspect ratio and circularity (Figure 4C–D), and reduced deformability (Figure 4E). LPC-exposed hRBCs became almost perfectly spherical at high concentration of LPC (Figure 4C). Retention rates of LPC-exposed hRBCs in the microspheres increased with the extent of surface area loss such that hRBCs with an average surface area loss >14% were unable to traverse the column of microspheres (Figure 4F). Given that the human RBC has an excess surface area of about 40% in relation to its volume [16], the present findings imply that an approximately 14% surface area loss is sufficient to affect RBC’s ability to deform and squeeze through the inter-microsphere spaces of the microsphilters that mimic human splenic IES.

## Discussion

This study shows that ring-iRBCs display a reduced membrane surface area and increased sphericity. This combination of parameters led to reduced deformability and retention of the ring-iRBCs in microsphilters. In contrast, co-cultured uRBCs displaying a limited surface area loss with unaltered sphericity and normal deformability were not retained in microsphilters. Trophozoite-iRBCs, which were poorly deformable in part due to a large and rigid parasite inside the host [9] were fully retained in the microsphilters.

Our study confirms the reduced surface area of ring-iRBCs documented using other experimental approaches [9], [10], [11]. The cell volume changes were not evaluated in the present study. Previous studies, however, found an increased [9], [10] or unchanged [11] cell volume for ring-iRBCs. Thus, the sphericity increase of ring-iRBCs that we observed might be the result of surface area loss documented by us and others [9], [10], [11] in conjunction with reported increased or constant cell volume [9], [10], [11].

The estimated values of surface area loss (17%, 15–18%) and sphericity increase (8%, 5–10%) of the retained ring-iRBCs are in line with the threshold of the LPC-exposed hRBCs retention in the microspheres (surface area loss and sphericity increase: >14%) or in the human spleen (surface area loss and sphericity increase: >18%) [8]. Taken together, these results outline that the combination of cell surface area loss and increased sphericity (i.e reduced S/V ratio) plays the determinant role in retention of ring-iRBCs in the microsphilters and in the spleen, and as such accounts for the difference between flow-through and retained ring-iRBCs. This result was suggested by a recent finding in a single malaria patient by another group using different approach showing that cell geometry might contribute to the splenic entrapment of ring-iRBCs [17]. The impaired deformability of a substantial fraction of ring-iRBCs hampers their ability to squeeze across the narrow and short inter-microbead spaces of the microsphilters [5].

Our data indicate that heterogeneity of culture-derived ring-iRBC morphometric characteristics drives their partitioning into flow-through and retained subpopulations. The non overlapping distribution of the various parameters assessed using Imagestream for the flow-through and upstream ring-iRBCs is consistent with two distinct subsets in approximately similar proportion in the culture, one displaying quasi-normal values, the other one displaying altered values. To formally prove this conclusion we need to improve the methodology to recover the ring-iRBCs retained in the microsphilters.

What is the basis for size and shape heretogeneity of synchronous ring-iRBCs? It could be of parasite origin, RBC origin (i.e., heterogeneity in the RBC population preexisting to *P. falciparum* merozoite invasion), or both. Three hypotheses could be considered.

Firstly, a range of parasite age, as the degree of alteration (surface area loss and shape change) increases with parasite maturation [9], [10], [11]. This however is unlikely, as the retention rates observed when very young rings (the oldest parasites being <6 hours post-invasion, with most parasites <4 hours post-invasion) were perfused through microsphilters or human spleens [5] were no different from the retention of older rings.

Secondly, the age of the host RBC might contribute to partitioning of the ring-iRBC population. RBC populations in the circulation contain cells with a wide age range (1 to 120 days). There is a loss surface area during RBC aging [18] resulting in a wide distribution of RBCs with varying extents of surface area loss, with older cells displaying approximately 20% surface area loss compared to younger RBCs [18]. Surface area loss is accompanied by a proportional loss in cell volume, so that the S/V ratio remains essentially constant during aging [18], thus avoiding massive physical trapping in the narrower blood vessels of the vascular bed or upstream the spleen red pulp littoral cells [19]. This also occurs *in vitro* when RBCs are co-cultured with *P. falciparum* (Figure 1D) [9], [10]. We can hypothesize that upon *P. falciparum* infection, the fraction of aging *Pf*-iRBCs will display more profound surface area loss and morphological changes compared to the fraction of younger *Pf*-iRBCs, and thus will be more likely to be retained in the microspheres. Additional investigations are needed to explore the actual contribution of RBC age on morphometric alterations of *P. falciparum* ring forms.

A third hypothesis would be that the flow-through ring-iRBCs might in contrast result from a restored cell size and S/V ratio of a fraction of ring-iRBCs. This phenomenon could be brought about by two processes, acquisition of membrane lipids extending their surface area [20] and/or modification of RBC ions and water permeability [21], [22], [23], [24], both resulting in restoration of the cell S/V ratio [8], [14] and preventing ring-iRBC retention in the microspheres. The restoration of the surface area is unlikely in our experiments, as the perfusion medium (Kreb’s-Albumax) used did not contain serum, and fat-free albumin was used. However, we cannot exclude that modification of RBC ions and water permeability could occur in a fraction of ring-iRBCs.

An additional, not mutually exclusive hypothesis would be that heterogeneity of ring-iRBCs results from heterogeneity in the quantity and possibly type of parasite products delivered to the host cell membrane upon merozoite invasion and early RBC remodeling [25], [26]. Microneme, rhoptry and dense granule proteins are delivered to the RBC membrane and cortical cytoskeleton [25], [26] and there is probably some heterogeneity in the type of proteins whose expression is regulated by epigenetic mechanisms (such as the microneme proteins from Erythrocyte Binding Ligands family and the rhoptry proteins from the Reticulocyte Binding Homolog family) at the population level, as well as possible heterogeneity in the amount of proteins delivered and/or engaging in stable interactions with the cortical cytoskeleton [25], [27].

In conclusion, we show the first direct demonstration that ImageStream technology can be used to analyze *P. falciparum*-infected red blood cell’s geometry. We show that RBCs hosting *P. falciparum* ring stage display a reduced surface area and increased sphericity. These properties are associated with their reduced deformability and retention in the microspheres. These findings provide the direct demonstration that these changes in RBC geometry drive the mechanical retention in microsphilters and as such contribute to entrapment of ring-iRBC in the human spleen. They further imply that estimation of total parasite burden in infected individuals based on number of circulating ring forms may underestimate the actual parasite burden.

## Materials and Methods

### Parasite Culturing

*Plasmodium falciparum* FUP-CB- alias FCR3- [28] was cultured as previously described [29]. Panning on human amelanotic C32 melanoma cells [30] was repeated until a cytoadherence rate of more than 5 iRBCs per C32 cell was obtained. Cytoadherent iRBCs were cultured in O+ erythrocytes (from blood center, Etablissement Français du Sang, Rungis) in RPMI 1640 medium containing bicarbonate and glutamine, 0.2% glucose, 50 µM hypoxanthine, 10 µg/mL gentamicin and 10% human AB+ serum, at 37°C in a humidified atmosphere containing 1% O_2_, 3% CO_2_ and 96% N_2_. Cultures were synchronized by successive multiple gel flotation and sorbitol (5%) treatments until the parasites reinvaded erythrocytes within 4 hours.

### Treatment of Red Blood Cells with Lysophosphatidylcholine

Blood from blood center (Etablissement Français du Sang, Rungis) was washed three times in RPMI 1640 to remove white blood cells. The treatment of RBCs was done as previously described [8]. Briefly, PKH-labeled RBCs [1] were resuspended in LPC (1 to 18 µmol/L) in phosphate buffer saline or PBS at 1% hematocrit to induce a controlled loss of membrane surface area. One of the critical parameters of this treatment is a low hematocrit (1%) needed to ensure homogeneity of the treated population. The LPC samples were incubated for 5 minutes at room temperature. Following incubation, samples were washed three times with PBS and resuspended in Krebs-albumin for further analysis.

### Measurement of RBC Deformability

RBC deformability was measured by ektacytometry using a laser-assisted optical rotational cell analyzer (LORCA; Mechatronics, Hoorn, The Netherlands) as previously described [1], [8], [31]. The unit of RBC deformability, namely, the elongation index (EI), was defined as the ratio between the difference between the 2 axes of the ellipsoid diffraction pattern and the sum of these 2 axes. RBC deformability was assessed over a range of shear stresses (0.3–30 Pa).

### Microsphere Filtration Process

Filtration of RBCs on microspheres was performed as described [5]. Briefly, calibrated metal micro-beads (Industrie des poudres Sphériques) with 2 different size distributions (5- to 15- µm-diameter and 15- to 25-µm-diameter) each from a single batch were used throughout. 2 g of dry micro-beads of each caliber was mixed and suspended in 8 mL Krebs, 1% AlbuMAX II (Invitrogen) and 600 µL of the bead suspension was poured into an inverted 1000-µL antiaerosol pipette tip (Neptune, BarrierTips) and allowed to settle, leading to the formation of a 5-mm-thick bead layer above the antiaerosol filter. 600 µL of a 2% hematocrit RBC suspension containing less than 10% of infected or potentially “retainable” LPC-treated RBCs (to avoid bead saturation) was introduced upstream from the micro-bead layer. Cells were perfused through the bead layer at a flow rate of 60 mL/h using an electric pump (Syramed µsp6000, Arcomed’Ag). The bead layer was then washed with 8 mL of Krebs/1%AlbuMAX II. The downstream sample was retrieved for further analysis.

### Analysis of Red Blood Cell Morphology and Dimensions

Cells were fixed with PBS-paraformaldehyde (1%) for analysis. Image acquisition and data analysis were done as described [8]. Briefly, images were acquired on the Imagestream® imaging cytometer (Ideas v4.0, Amnis Corp., Seattle, WA). At least 10,000 images were collected for each sample. Post-acquisition data analysis was performed using IDEAS® image analysis software package (Inspire v4.0, Amnis Corp.). Morphological (circularity and aspect ratio) and dimension (projected surface area, diameter and perimeter) features of red cells (*Pf*-iRBCs or uninfected RBCs) were calculated using bright filter images of RBC by IDEAS software. Imagestream technology will estimate one half the total cell surface area (projected surface area), since one-side of the cell’s image is captured. In order to compare the cell surface area between the different populations of RBCs, we defined a parameter named cell “surface area loss” which is the percentage of “projected surface area loss”. The “projected surface area loss” of *Pf*-iRBCs or uRBCs was defined with this formula: [1– (mean values of projected surface area of iRBCs or uRBCs/mean values of projected surface area of hRBC)]*100. One can be robustly assume that the total cell surface area = K*(cell projected surface area), K being a constant value. The proportion of “projected surface area loss” and “surface area loss” are identical.

Aspect ratio will measure ratios of cell’s diameters. Since we were unable to measure total cell surface area and volume, we used cell’s aspect ratio as an accurate parameter to evaluate cell’s sphericity based on the results of previous findings [8]. The more aspect ratio (AS) values tend to 1, the more the cell is spherical [8]. In order to compare cell’s sphericity between the different populations of RBCs, we defined a parameter named cell “sphericity increase”. The sphericity increase of uRBCs or *Pf*-iRBCs was defined with this formula: [(AS value of uRBCs or iRBCs/mean AS values of hRBC) –1]*100.

### Osmotic Fragility Test

Osmotic fragility of RBC was determined according to the method originally described by Parpart et al. [32]. Cells were incubated during 30 minutes in hypotonic solutions with NaCl content ranging from 0.1% to 0.9% (hematocrit: 1%). After centrifugation, absorbance of the supernatants was measured at 540 nm using a spectrophotometer and the percent hemolysis was calculated for each supernatant and plotted against NaCl concentration. The resulting osmotic fragility curves of parasitized or LPC-treated RBC were then compared with that obtained with normal controls.

### Statistical Analysis

We used the Student paired t test or OneWay ANOVA with a Tukey posthoc analysis for statistical analysis; p values less than 0.05 were considered statistically significant. All statistical analyses were performed using SPSS statistical software (PASW statistic version 18).
